# Statistical data analysis of cancer incidences in insurgency affected states in Nigeria

**DOI:** 10.1016/j.dib.2018.04.135

**Published:** 2018-05-05

**Authors:** Patience I. Adamu, Pelumi E. Oguntunde, Hilary I. Okagbue, Olasunmbo O. Agboola

**Affiliations:** Department of Mathematics, Covenant University, Ota, Ogun State, Nigeria

**Keywords:** Cancer, Chi-square test of independence, Insurgency, Nigeria, Regression model, Statistics

## Abstract

This article provides details about the various cancer types recorded in Northeastern states of Nigeria currently being affected by insurgency in Nigeria. The dataset was described and chi-square test was used to determine the dependency of the variables under consideration on each other. Also, linear, logarithmic, inverse, quadratic, cubic, power, growth, exponential and logistic regression models were fitted to the dataset to show the relationship between them.

**Specifications Table**

**Table:** 

Subject area	Medicine
More specific subject area	Oncology, Public health, Biostatistics
Type of data	Table and text file
How data was acquired	Secondary data from University of Maiduguri Teaching Hospital.
Data format	Raw and partially analyzed (Descriptive and Inferential)
Experimental factors	Analysis of cancer incidences
Experimental features	Observations on the age, gender and the topographical location of cancer on the body of affected patients
Data source location	University of Maiduguri Teaching hospital, Maiduguri, Borno state, Northeast Nigeria.
Data accessibility	All the data are available this article

**Value of the data**

• The data is useful in the study of epidemiology of cancer in the affected areas.
• The data is an indication of the public health crisis in insurgency affected region in Nigeria.
• The data can be useful in cancer awareness, management and treatment.
• The data could be used in oncologic studies.
• The data can be used to test the performance of statistical models.

## 1 Data

The data set represents the age, gender and topological (Top) location of cancer on the body of cancer patients in the University of Maiduguri Teaching hospital located in Maiduguri, the capital of Borno state, Nigeria.

The teaching hospital is the only tertiary health care facility in the state and often serves the other northeast states like Yobe, Taraba, Adamawa, Bauchi and Gombe.

A total of 1671 patients were considered between the period of study and SPSS version 20 was used to perform the analysis. The dataset is available as Supplementary data while a brief summary of the data is presented in Table 1.

**Table 1:** Brief summary of the data.

**Statistics**				
		Gender	Age	Top
N	Valid	1670	1671	1671
	Missing	1	0	0
Mean		1.53	50.06	37.59
Mode		2	60	5
Variance		0.249	281.086	816.431
Skewness		−0.115	−0.258	0.241
Std. Error of Skewness		0.060	0.060	0.060
Kurtosis		−1.989	−0.220	−1.149
Std. Error of Kurtosis		0.120	0.120	0.120
Minimum		1	3	1
Maximum		2	95	117
Sum		2553	83,658	62,806

It was observed from Table 1 that information about the gender of a patient was not available, hence the missing data of 1.

The frequency distribution of the gender of the patients is presented in Table 2.

**Table 2:** Frequency distribution of the patients’ gender.

Gender		Frequency	Percent	Cumulative Percent
Valid	Male	787	47.1	47.1
	Female	883	52.8	100.0
	Total	1670	99.9	
Missing	System	1	.1	
Total		1671	100.0	

**Fig. 1:**
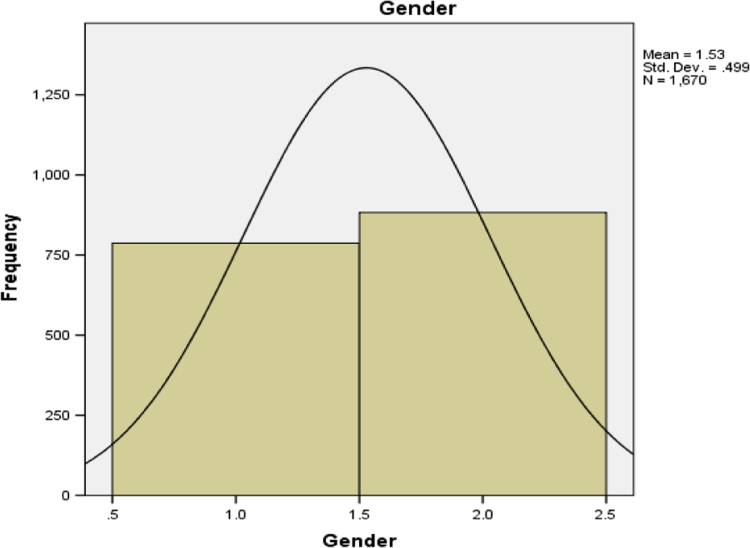
Gender of the patients.

**Remark:**Table 2 indicates that there are more female patients with cancer diseases than males. This is represented in a pictorial form in Fig. 1.

The frequency distribution of the patients’ age is presented in Table 3.

**Table 3:** Frequency distribution of the patient's age.

Age (years)	Frequency	Percent	Cumulative Percent
3	6	0.4	0.4
4	5	0.3	0.7
5	1	0.1	0.7
6	5	0.3	1.0
7	5	0.3	1.3
8	2	0.1	1.4
9	1	0.1	1.5
10	2	0.1	1.6
12	4	0.2	1.9
14	4	0.2	2.1
15	8	0.5	2.6
16	6	0.4	2.9
17	4	0.2	3.2
18	9	0.5	3.7
19	6	0.4	4.1
20	15	0.9	5.0
22	9	0.5	5.5
23	12	0.7	6.2
24	11	0.7	6.9
25	17	1.0	7.9
26	11	0.7	8.6
27	15	0.9	9.5
28	19	1.1	10.6
29	7	0.4	11.0
30	51	3.1	14.1
31	6	0.4	14.4
32	22	1.3	15.7
33	7	0.4	16.2
34	10	0.6	16.8
35	74	4.4	21.2
36	16	1.0	22.1
37	15	0.9	23.0
38	27	1.6	24.7
39	13	0.8	25.4
40	94	5.6	31.1
41	13	0.8	31.8
42	18	1.1	32.9
43	15	0.9	33.8
44	11	0.7	34.5
45	74	4.4	38.9
46	18	1.1	40.0
47	13	0.8	40.8
48	32	1.9	42.7
49	11	0.7	43.3
50	134	8.0	51.3
51	12	0.7	52.1
52	23	1.4	53.4
53	19	1.1	54.6
54	23	1.4	56.0
55	94	5.6	61.6
56	26	1.6	63.1
57	18	1.1	64.2
58	19	1.1	65.4
59	7	0.4	65.8
60	161	9.6	75.4
61	9	0.5	75.9
62	13	0.8	76.7
63	9	0.5	77.3
64	8	0.5	77.7
65	82	4.9	82.6
66	6	0.4	83.0
67	10	0.6	83.6
68	16	1.0	84.6
69	2	0.1	84.7
70	128	7.7	92.3
71	5	0.3	92.6
72	8	0.5	93.1
73	4	0.2	93.4
74	3	0.2	93.5
75	26	1.6	95.1
76	5	0.3	95.4
77	5	0.3	95.7
78	6	0.4	96.1
79	2	0.1	96.2
80	36	2.2	98.3
81	1	0.1	98.4
82	1	0.1	98.4
83	1	0.1	98.5
84	2	0.1	98.6
85	13	0.8	99.4
86	2	0.1	99.5
90	6	0.4	99.9
93	1	0.1	99.9
95	1	0.1	100.0
Total	1671	100.0	

**Fig. 2:**
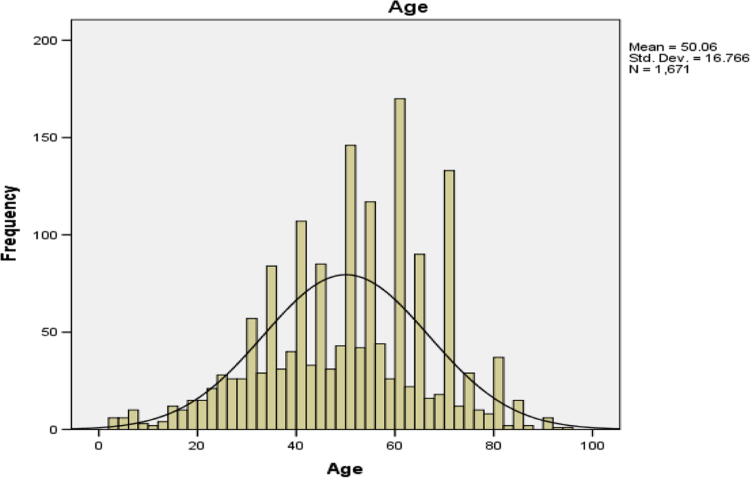
Age of the patients.

**Remarks:** From Table 3, the lowest age captured is 3 years old while the oldest patient is 95 years old. The cancer diseases affected both young and old but particularly, the age of the patients with highest number of cancer incidence is 60 years old. This information is represented in Fig. 2.

The various parts of the body affected by cancer incidences and the number of people affected (frequencies) are indicated in Table 4.

**Table 4:** Parts of the body affected by the various types of cancer.

**Topological (Top) location of cancer**		Frequency	Percent	Cumulative Percent
Valid	C77.9 Lymph node, NOS	9	0.5	0.5
	C26.9 Gastrointestinal tract, NOS	9	0.5	1.1
	C20.9 Rectum, NOS	54	3.2	4.3
	C44.9 Skin, NOS	47	2.8	7.1
	C61.9 Prostate gland	253	15.1	22.3
	C63.9 Male genital organs, NOS	1	0.1	22.3
	C49.6 Soft tissues of trunk	5	0.3	22.6
	C50.9 Breast, NOS	92	5.5	28.1
	C77.3 Lymph nodes of axilla or arm	2	0.1	28.2
	C57.9 Female genital tract, NOS	15	0.9	29.1
	C53.9 Cervix uteri	76	4.5	33.7
	C22.0 Liver	31	1.9	35.5
	C77.0 Lymph nodes of head, face and	6	0.4	35.9
	C40.9 Bone of limb, NOS	4	0.2	36.1
	C53.8 Overl. lesion of cervix uteri	1	0.1	36.2
	C49.2 Soft tissues of lower limb an	7	0.4	36.6
	C49.9 Other soft tissues	18	1.1	37.7
	C67.9 Urinary bladder, NOS	32	1.9	39.6
	C56.9 Ovary	60	3.6	43.2
	C40.2 Long bones of lower limb	1	0.1	43.3
	C44.2 External ear	1	0.1	43.3
	C49.0 Soft tissues of head, face, &	9	0.5	43.9
	C44.7 Skin of lower limb and hip	6	0.4	44.2
	C39.9 Ill-defined sites within resp	15	0.9	45.1
	C49.1 Soft tissues of upper limb, s	4	0.2	45.4
	C44.6 Skin of upper limb and shoulder	3	0.2	45.5
	C19.9 Rectosigmoid junction	4	0.2	45.8
	C64.9 Kidney, NOS	20	1.2	47.0
	C40.8 Overl. lesion of bones of lim	1	0.1	47.0
	C41.0 Bones of skull and face	2	0.1	47.2
	C44.4 Skin of scalp and neck	6	0.4	47.5
	C16.3 Gastric antrum	6	0.4	47.9
	C18.0 Cecum	20	1.2	49.1
	C16.9 Stomach, NOS	7	0.4	49.5
	C49.5 Soft tissues of pelvis	3	0.2	49.7
	C04.9 Floor of mouth, NOS	2	0.1	49.8
	C73.9 Thyroid gland	14	0.8	50.6
	C77.1 Intrathoracic lymph nodes	1	0.1	50.7
	C52.9 Vagina, NOS	8	0.5	51.2
	C10.2 Lateral wall of oropharynx	1	0.1	51.2
	C44.5 Skin of trunk	2	0.1	51.3
	C69.0 Conjunctiva	14	0.8	52.2
	C21.8 Overl. lesion rectum, anal ca	9	0.5	52.7
	C49.4 Soft tissues of abdomen	4	0.2	53.0
	C18.4 Transverse colon	1	0.1	53.0
	C41.9 Bone, NOS	1	0.1	53.1
	C76.2 Abdomen, NOS	1	0.1	53.1
	C76.5 Lower limb, NOS	1	0.1	53.2
	C69.6 Orbit, NOS	1	0.1	53.3
	C49.3 Soft tissues of thorax	3	0.2	53.4
	C55.9 Uterus, NOS	30	1.8	55.2
	C44.8 Overl. lesion of skin	1	0.1	55.3
	C51.9 Vulva, NOS	1	0.1	55.4
	C10.9 Oropharynx, NOS	2	0.1	55.5
	C30.1 Middle ear	1	0.1	55.5
	C62.9 Testis, NOS	2	0.1	55.7
	C15.0 Cervical esophagus	12	0.7	56.4
	C18.7 Sigmoid colon	1	0.1	56.4
	C80.9 Unknown primary site	200	12.0	68.4
	C77.2 Intra-abdominal lymph nodes	1	0.1	68.5
	C11.9 Nasopharynx, NOS	3	0.2	68.6
	C50.0 Nipple	168	10.1	78.7
	C53.0 Endocervix	105	6.3	85.0
	C53.1 Exocervix	1	0.1	85.0
	C67.4 Posterior wall of urinary bla	8	0.5	85.5
	C16.0 Cardia, NOS	33	2.0	87.5
	C21.0 Anus, NOS	17	1.0	88.5
	C51.0 Labium majus	3	0.2	88.7
	C67.0 Trigone of urinary bladder	57	3.4	92.1
	C44.0 Skin of lip, NOS	15	0.9	93.0
	C11.0 Superior wall of nasopharynx	16	1.0	94.0
	C08.0 Submandibular gland	3	0.2	94.1
	C14.0 Pharynx, NOS	5	0.3	94.4
	C26.0 Intestinal tract, NOS	7	0.4	94.9
	C65.9 Renal pelvis	4	0.2	95.1
	C10.0 Vallecula	6	0.4	95.5
	C25.0 Head of pancreas	5	0.3	95.8
	C60.0 Prepuce	4	0.2	96.0
	C21.2 Cloacogenic zone	4	0.2	96.2
	C18.6 Descending colon	1	0.1	96.3
	C66.9 Ureter	1	0.1	96.3
	C50.1 Central portion of breast	1	0.1	96.4
	C34.0 Main bronchus	1	0.1	96.5
	C21.1 Anal canal	3	0.2	96.6
	C18.9 Colon, NOS	1	0.1	96.7
	C01.9 Base of tongue, NOS	3	0.2	96.9
	C62.0 Undescended testis	4	0.2	97.1
	C11.2 Lateral wall of nasopharynx	1	0.1	97.2
	C50.6 Axillary tail of breast	1	0.1	97.2
	C54.1 Endometrium	2	0.1	97.4
	C25.9 Pancreas, NOS	1	0.1	97.4
	C30.0 Nasal cavity	1	0.1	97.5
	C00.9 Lip, NOS	1	0.1	97.5
	C54.2 Myometrium	1	0.1	97.6
	C48.8 Overl. lesion of retroperiton	1	0.1	97.7
	C76.7 Other ill-defined sites	1	0.1	97.7
	C03.0 Upper gum	2	0.1	97.8
	C15.9 Oesophagus, NOS	1	0.1	97.9
	C69.9 Eye, NOS	1	0.1	98.0
	C16.4 Pylorus	1	0.1	98.0
	C07.9 Parotid gland	2	0.1	98.1
	C67.5 Bladder neck	1	0.1	98.2
	C57.4 Uterine adnexa	1	0.1	98.3
	C16.2 Body of stomach	1	0.1	98.3
	C13.0 Postcricoid region	7	0.4	98.7
	C37.9 Thymus	1	0.1	98.8
	C17.0 Duodenum	1	0.1	98.9
	C06.0 Cheek mucosa	1	0.1	98.9
	C04.0 Anterior floor of mouth	4	0.2	99.2
	C47.0 Per. nerves & A.N.S. of head,	3	0.2	99.3
	C09.0 Tonsillar fossa	2	0.1	99.5
	C38.4 Pleura, NOS	1	0.1	99.5
	C38.0 Heart	4	0.2	99.8
	C67.1 Dome of urinary bladder	1	0.1	99.8
	C22.1 Intrahepatic bile duct	1	0.1	99.9
	C76.0 Head, face or neck, NOS	1	0.1	99.9
	C23.9 Gallbladder	1	0.1	100.0
	Total	1671	100.0	

**Fig. 3:**
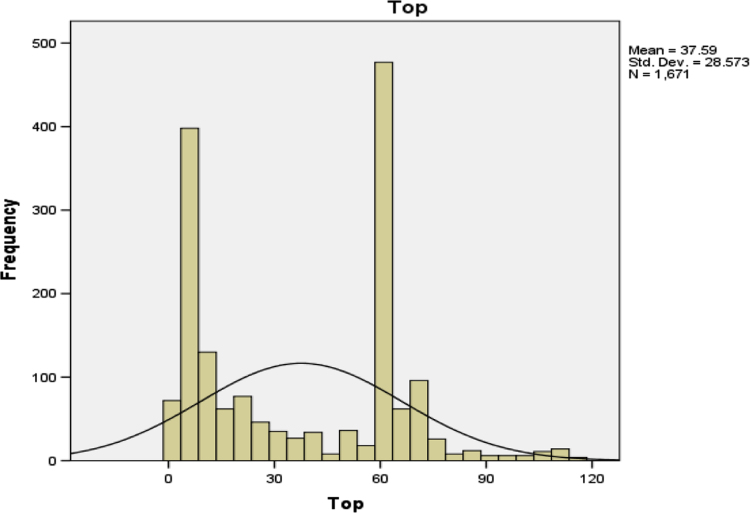
Diagrammatic presentation of the parts of the body affected by cancer.

Table 4 shows that the part of the body affected mostly is the prostate gland. This is represented graphically in Fig. 3.

## 2 Experimental design, materials and methods

The data set was obtained from the patients’ records at the data center of the University of Maiduguri teaching hospital. The hospital as stated earlier serves a large population from the six Northeastern states of Nigeria and beyond. The Northeastern region in particular and the entire northern region of the country is in variance with their natural endowments such as vast fertile lands, rivers and lakes for irrigation, mineral resources and abundant sunshine for renewable energy. The weak social structure of the region has resulted to excruciating poverty which often manifest as homelessness and destitution, insurgency, violence and crime [1]. The region has high poverty index, low human development index, lack of portable drinking water, electoral violence, dearth of medical personnel, high mortality, low life expectancy, decayed infrastructure and also an epicenter for joblessness, underage and teenage pregnancy, female genital mutilation, epidemics, illiteracy, malnutrition and now terrorism which comes in form of coordinated attacks on military, police formations and remote villages, guerrilla attacks, kidnappings, regicide, suicide bombings, mass killings, abduction of school girls, extra-judicial killings and summary execution, hypnotizing and forced conscriptions, indoctrination and forceful conversion to Islam and so on. The decadence is assumed to be as a result of corruption, tribalism, military intervention in governance, inequality, misappropriation, financial recklessness, bankrupt of ideas and dearth of developmental agendas, reduction of allocation of capital due to shortfalls of Nigeria revenue as a result of decline in crude oil price. Globally, efforts towards improving the healthcare and reducing the incidence of cancer have yielded desired results except in some developing countries. Hence, cancer related deaths remain stubbornly high in those countries. Cancer awareness, screening, prevention, management, treatment strategies are very low in the region/area studied in this article. Regrettably, capital allocations to the health sector are inadequate and the available funds are often allegedly diverted by corrupt government officials.

In addition, maternal death is one area that is currently affected by the Boko haram insurgency in that region as reported by [2]. Moreover, other areas have been seriously affected; for example; food security and dynamics, under five malnutrition, child mortality, escalation of cholera outbreaks, infections, sexually transmitted diseases, unsafe birth practices and abortion, child prostitution, sex for food at the displaced persons camps, increase in polio cases, See [3], [4], [5], [6], [7], [8] for details. Some related article can also be explored [9], [10], [11], [12], [13], [14], [15], [16], [17], [18], [19], [20], [21], [22], [23], [24], [25], [26], [27], [28], [29], [30], [31].

Next, we analyze the dataset collected using Chi-square test of independence and curve estimation.

### 2.1 Chi-square test of independence

Chi-square test of independence was used to investigate the relationship between the location of the cancer (top), gender and age of patients.

#### 2.1.1 Test of independency between “Top” and gender of the patients

Hypothesis Testing I:

H_0_: There is no significant association between the topological location of cancer and the gender of the patients.

Versus.

H_1_: There is a significant association between the topological location of cancer and the gender of the patients.

The result of the analysis is presented in Table 5.

**Table 5:** Result of the chi-square test between gender and “Top”.

**Chi-Square Tests**	Value	df	Asymp. Sig. (2-sided)
Pearson Chi-Square	928.735	116	0.000
Likelihood Ratio	1214.083	116	0.000
Linear-by-Linear Association	64.659	1	0.000
N of Valid Cases	1670		

**Remarks:** The null hypothesis (H_0_) is rejected since the *p*-value (0.000) is less than the level of significance (0.05). Therefore, it can be concluded that there is a significant association between the topological location of cancer and the gender of the patients.

The information about the correlation coefficient and its corresponding *p*-value is presented in Table 6.

**Table 6:** Correlation coefficient.

**Symmetric Measures**		Value	Asymp. Std. Error	Approx. *T*	Approx. Sig.
Interval by Interval	Pearson's *R*	0.197	0.024	8.199	0.000
Ordinal by Ordinal	Spearman Correlation	0.253	0.024	10.661	0.000
N of Valid Cases		1670			

#### 2.1.2 Test of independency between “Top” and age of the patients

Hypothesis Testing II:

H_0_: There is no significant association between topological location of cancer is not dependent on the age of the patients.

Versus.

H_1_: There is a significant association between topological location of cancer is dependent on the age of the patients.

The result of the analysis is presented in Table 7.

**Table 7:** Result of the chi-square test between age and “Top”.

**Chi-Square Tests**	Value	Df	Asymp. Sig. (2-sided)
Pearson Chi-Square	10762.735	9628	0.000
Likelihood Ratio	3148.516	9628	1.000
Linear-by-Linear Association	50.758	1	0.000
N of Valid Cases	1671		

**Remarks:** Since the *p*-value is also less than 0.05, we conclude that there is a significant association between the topological location of cancer and the age of the patients.

Information about the correlation coefficient and its corresponding *p*-value is presented in Table 8.

**Table 8:** Correlation coefficient result.

**Symmetric Measures**		Value	Asymp. Std. Error	Approx. *T*	Approx. Sig.
Interval by Interval	Pearson's R	−.174	0.024	−7.233	0.000
Ordinal by Ordinal	Spearman Correlation	−.189	0.025	−7.881	0.000
N of Valid Cases		1671			

### 2.2 Curve estimation

Linear, logarithmic, inverse, quadratic, cubic, power, growth, exponential and logistic regression models were fitted to the dataset. “Top” is the dependent variable while Age is the independent variable. The summary of the variables used is presented in Table 9.

**Table 9:** Summary of the variables.

**Variable Processing Summary**		Variables	
		Dependent	Independent
		Top	Age
Number of Positive Values		1671	1671
Number of Zeros		0	0
Number of Negative Values		0	0
Number of Missing Values	User-Missing	0	0
	System-Missing	0	0

#### 2.2.1 Simple linear regression

The summary of the simple linear regression model is presented in Table 10.

**Table 10:** Model summary.

*R*	*R* Square	Adjusted *R* Square	Std. Error of the Estimate
0.174	0.030	0.030	28.144

The independent variable is Age.

The corresponding analysis of variance (ANOVA) table testing for the fitness of the model is presented in Table 11.

**Table 11:** ANOVA table for the linear model.

**ANOVA**					
	Sum of Squares	df	Mean Square	*F*	Sig.
Regression	41440.679	1	41440.679	52.318	0.000
Residual	1321998.748	1669	792.090		
Total	1363439.427	1670			

The independent variable is Age.

The linear regression model is significant at 0.05 level of significance and with *R*-square value of 3%.

#### 2.2.2 Logarithmic model

The summary of the logarithmic model is presented in Table 12.

**Table 12:** Model summary for the logarithmic model.

*R*	*R* Square	Adjusted *R* Square	Std. Error of the Estimate
0.130	0.017	0.016	28.340

The independent variable is Age.

Estimating the model parameter gives the result in Table 13.

**Table 13:** Parameter estimation for the logarithmic model.

**Coefficients**	Unstandardized Coefficients		Standardized Coefficients	*T*	Sig.
	*B*	Std. Error	Beta		
ln(Age)	−8.130	1.520	−0.130	−5.349	0.000
(Constant)	68.755	5.869		11.716	0.000

The ANOVA table for the logarithmic model is presented in Table 14.

**Table 14:** ANOVA table for the logarithmic model.

**ANOVA**					
	Sum of Squares	df	Mean Square	*F*	Sig.
Regression	22977.216	1	22977.216	28.609	0.000
Residual	1340462.210	1669	803.153		
Total	1363439.427	1670			

The independent variable is Age.

The logarithmic model is significant at 0.05 level of significance and with R-square value of 1.7%.

#### 2.2.3 Inverse model

The summary of the inverse model is presented in Table 15.

**Table 15:** Summary of the inverse model.

*R*	*R* Square	Adjusted *R* Square	Std. Error of the Estimate
0.047	0.002	0.002	28.550

The independent variable is age.

The result for the estimation of parameters using the inverse model is presented in Table 16.

**Table 16:** Parameter estimation using inverse model.

**Coefficients**	Unstandardized Coefficients		Standardized Coefficients	*t*	Sig.
	B	Std. Error	Beta		
1/Age	49.544	25.664	0.047	1.930	0.054
(Constant)	36.327	0.956		38.018	0.000

The corresponding ANOVA table is presented in Table 17.

**Table 17:** The ANOVA table for the inverse model.

**ANOVA**					
	Sum of Squares	df	Mean Square	*F*	Sig.
Regression	3037.719	1	3037.719	3.727	0.054
Residual	1360401.707	1669	815.100		
Total	1363439.427	1670			

The independent variable is age.

The inverse model is not significant as its *p*-value is greater than the level of significance (0.05).

#### 2.2.4 Quadratic model

The summary for the quadratic model is presented in Table 18.

**Table 18:** Summary for the quadratic model.

*R*	*R* Square	Adjusted *R* Square	Std. Error of the Estimate
0.195	0.038	0.037	28.043

The independent variable is age.

The result for the estimation of parameter using the quadratic model is presented in Table 19.

**Table 19:** Parameter estimation for the quadratic model.

**Coefficients**	Unstandardized Coefficients		Standardized Coefficients	*t*	Sig.
	*B*	Std. Error	Beta		
Age	0.348	0.183	0.204	1.897	0.058
Age ** 2	−0.007	0.002	−0.388	−3.607	0.000
(Constant)	38.929	4.329		8.992	0.000

The corresponding ANOVA table is presented in Table 20.

**Table 20:** ANOVA table for the quadratic model.

**ANOVA**					
	Sum of Squares	df	Mean Square	*F*	Sig.
Regression	51674.289	2	25837.144	32.854	0.000
Residual	1311765.138	1668	786.430		
Total	1363439.427	1670			

The independent variable is age.

The quadratic model is significant at 0.05 level of significance and with *R*-square value of 3.8%.

#### 2.2.5 Cubic model

The summary for the cubic model is presented in Table 21.

**Table 21:** Summary for the cubic model.

*R*	*R* Square	Adjusted *R* Square	Std. Error of the Estimate
0.197	0.039	0.037	28.036

The independent variable is age.

The result for the estimation of parameter for the cubic model is presented in Table 22.

**Table 22:** Parameter estimation for the cubic model.

**Coefficients**	Unstandardized Coefficients		Standardized Coefficients	*t*	Sig.
	*B*	Std. Error	Beta		
Age	0.951	0.477	0.558	1.993	0.046
Age ** 2	−0.021	0.011	−1.230	−1.970	0.049
Age ** 3	0.000	0.000	0.504	1.369	0.171
(Constant)	32.108	6.601		4.864	0.000

The corresponding ANOVA table is presented in Table 23.

**Table 23:** ANOVA table for the cubic model.

**ANOVA**					
	Sum of Squares	df	Mean Square	*F*	Sig.
Regression	53146.668	3	17715.556	22.538	0.000
Residual	1310292.759	1667	786.018		
Total	1363439.427	1670			

The independent variable is age.

The cubic model is significant and with *R*-square value of 3.9%.

#### 2.2.6 Power model

The summary for the power model is presented in Table 24.

**Table 24:** Summary for the power model.

*R*	*R* Square	Adjusted *R* Square	Std. Error of the Estimate
0.159	0.025	0.025	1.125

The independent variable is age.

The result for the estimation of parameter for the power model is presented in Table 25.

**Table 25:** Parameter estimation for the power model.

**Coefficients**	Unstandardized Coefficients		Standardized Coefficients	*t*	Sig.
	*B*	Std. Error	Beta		
ln(Age)	−0.397	0.060	−0.159	−6.583	0.000
(Constant)	105.955	24.692		4.291	0.000

The dependent variable is ln(Top).

The corresponding ANOVA table is presented in Table 26.

**Table 26:** ANOVA table for the power model.

**ANOVA**					
	Sum of Squares	df	Mean Square	*F*	Sig.
Regression	54.875	1	54.875	43.330	0.000
Residual	2113.710	1669	1.266		
Total	2168.585	1670			

The independent variable is age.

The power model is significant at 0.05 level of significance and with R-square value of 2.5%.

#### 2.2.7 Growth model

The model summary for the growth model is presented in Table 27.

**Table 27:** Summary for the growth model.

*R*	*R* Square	Adjusted *R* Square	Std. Error of the Estimate
0.216	0.047	0.046	1.113

The independent variable is age.

The result for the estimation of parameter of the growth model is presented in Table 28.

**Table 28:** Parameter estimation for the growth model.

**Coefficients**	Unstandardized Coefficients		Standardized Coefficients	*t*	Sig.
	*B*	Std. Error	Beta		
Age	−0.015	0.002	−0.216	−9.038	0.000
(Constant)	3.875	0.086		45.180	0.000

The dependent variable is ln(Top).

The corresponding ANOVA table is presented in Table 29.

**Table 29:** ANOVA table for the growth model.

**ANOVA**					
	Sum of Squares	df	Mean Square	*F*	Sig.
Regression	101.181	1	101.181	81.683	0.000
Residual	2067.404	1669	1.239		
Total	2168.585	1670			

The independent variable is age.

The growth model is significant at 0.05 level of significance and with *R*-square value of 4.7%.

#### 2.2.8 Exponential model

The model summary for the exponential model is presented in Table 30.

**Table 30:** Summary for the exponential model.

*R*	*R* Square	Adjusted *R* Square	Std. Error of the Estimate
0.216	0.047	0.046	1.113

The independent variable is age.

The result for the estimation of parameter for the exponential model is presented in Table 31.

**Table 31:** Parameter estimation for the exponential model.

**Coefficients**	Unstandardized Coefficients		Standardized Coefficients	*t*	Sig.
	*B*	Std. Error	Beta		
Age	−0.015	0.002	−0.216	−9.038	0.000
(Constant)	48.173	4.132		11.660	0.000

The dependent variable is ln(Top).

The corresponding ANOVA table is presented in Table 32.

**Table 32:** ANOVA table for the exponential model.

**ANOVA**					
	Sum of Squares	df	Mean Square	*F*	Sig.
Regression	101.181	1	101.181	81.683	0.000
Residual	2067.404	1669	1.239		
Total	2168.585	1670			

The independent variable is age.

The exponential model is significant at 0.05 level of significance and with *R*-square value of 4.7%.

#### 2.2.9 Logistic model

The model summary for the logistic model is presented in Table 33.

**Table 33:** Summary for the logistic model.

*R*	*R* Square	Adjusted *R* Square	Std. Error of the Estimate
0.216	0.047	0.046	1.113

The independent variable is age.

The estimation of parameters for the logistic model is presented in Table 34.

**Table 34:** Parameter estimation for the logistic model.

**Coefficients**	Unstandardized Coefficients		Standardized Coefficients	*t*	Sig.
	*B*	Std. Error	Beta		
Age	1.015	0.002	1.241	615.592	0.000
(Constant)	0.021	0.002		11.660	0.000

The dependent variable is ln(1 / Top).

The corresponding ANOVA table is presented in Table 35.

**Table 35:** ANOVA table for the logistic model.

**ANOVA**					
	Sum of Squares	df	Mean Square	*F*	Sig.
Regression	101.181	1	101.181	81.683	0.000
Residual	2067.404	1669	1.239		
Total	2168.585	1670			

The independent variable is age.

The logistic model is also significant at 0.05 level of significance and with *R*-square value of 4.7%.

**Fig. 4:**
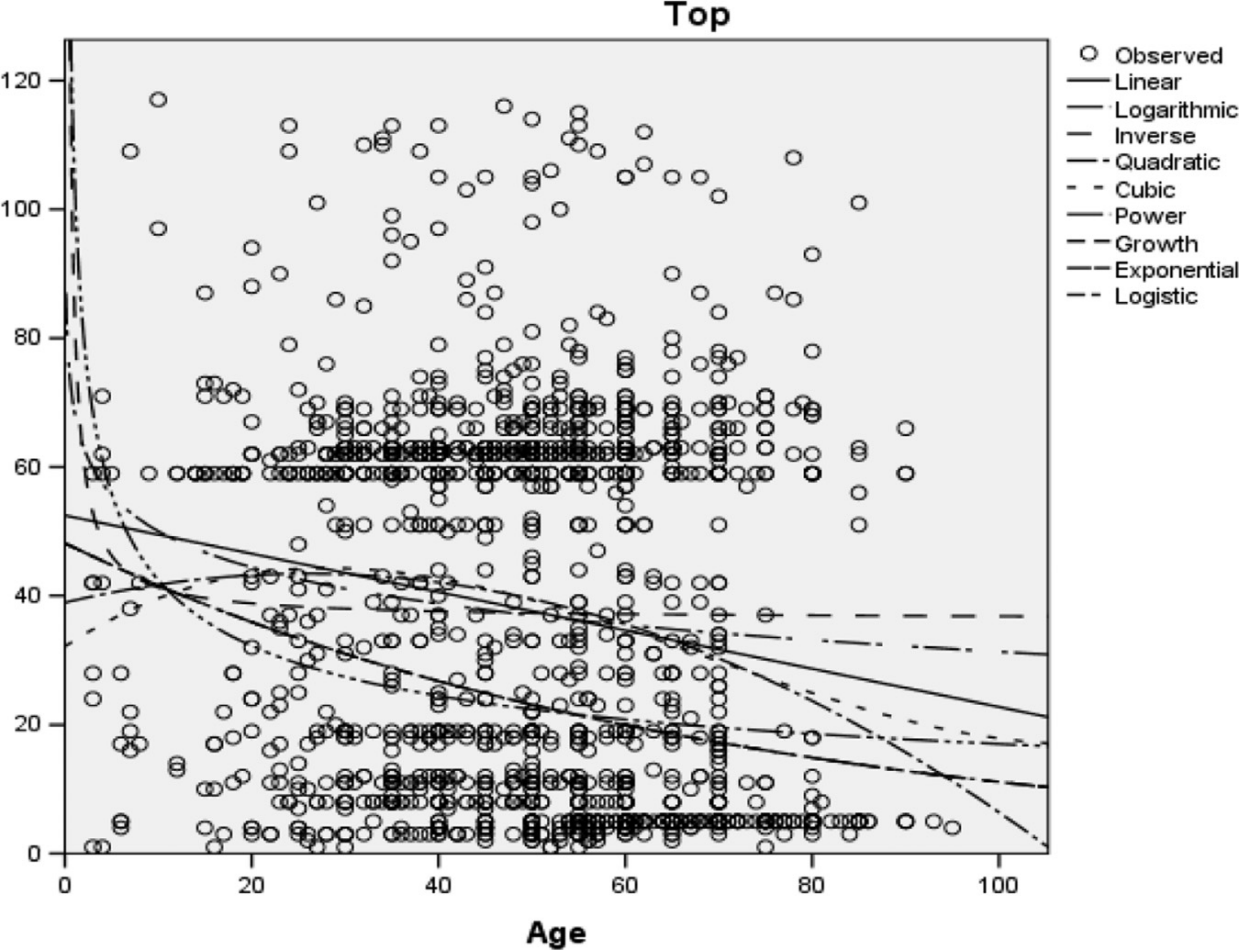
The fitted model with respect to the data set.

Lastly, all the fitted models are illustrated in Fig. 4.

**Important points**

• More females are infected with cancer than men.
• The age with the highest record (or incidence) of cancer is 60 years old.
• The part of the body that is mostly affected by cancer is the prostate gland (based on the data set collected).
• There is a significant association between the topological location of cancer and the gender of the patients.
• There is a significant association between the topological location of cancer and the age of the patients.
• All the models fitted to the data produced low *R*-square values; nevertheless, the models that best fit the data based on their *R*-square values are growth model, exponential model and logistic model.
