# Supplementary material for: Statistical data analysis of cancer incidences in insurgency affected states in Nigeria
Source: Data Brief. 2018 May 5;18:2029–46. doi: 10.1016/j.dib.2018.04.135 (PMC5998707; doi:10.1016/j.dib.2018.04.135)
Supplement: Supplementary file 1 — Supplementary material [file mmc1.pdf]

COVENANT UNIVERSITY

COLLEGE OF SCIENCE AND TECHNOLOGY

DEPARTMENT OF MATHEMATICS

CANAANLAND, KM 10, IDIROKO ROAD
P.M.B 1023, OTA, OGUN STATE, NIGERIA
www.covenantuniversity.edu.ng, mat.covenantuniversity.edu.ng

EXTERNAL MEMO

To: Editor, Data in Brief
From: Corresponding Author
Date: 18th April, 2018
Subject: **Conflict of Interest**

I thereby declare the absence of any conflict of interest among the authors.

The authors have read the final draft and unanimously agreed that the paper be sent for review.

The source of funding and data have been acknowledged.

Dr. Pelumi E. Oguntunde

pelumi.oguntunde@covenantuniversity.edu.ng

Department of Mathematics,

Covenant University, Ota, Nigeria
